# Data on interference indices in body fluid specimens submitted for clinical laboratory analysis

**DOI:** 10.1016/j.dib.2020.105408

**Published:** 2020-03-10

**Authors:** Renee L. Eigsti, Matthew D. Krasowski, Aditi Vidholia, Anna E. Merrill

**Affiliations:** Department of Pathology, University of Iowa Hospitals and Clinics, 200 Hawkins Drive, Iowa City, IA 52242, USA

**Keywords:** Assay interference, Body fluid analysis, Clinical chemistry, Hemolysis, Icterus, Lipemia

## Abstract

Clinical chemistry analysis of body fluids from non-blood or urine sources presents a technical challenge for clinical laboratories. Examples of body fluids include biliary secretions, cerebrospinal fluid, cyst contents, dialysate, gastric aspirates, peritoneal fluid, pleural fluid, stool, surgical drain fluid, synovial fluid, and wound exudates. The heterogeneous nature of these body fluids presents technical difficulties for analysis. For example, body fluid specimens may have presence of hemolysis, icterus, or lipemia (‘interference indices’) that can interfere with clinical chemistry analysis. In the related research article, we analyzed the distribution of these interference indices and body fluid samples submitted for analysis at an academic medical center central clinical laboratory and compared this to data from serum/plasma specimens. The data in this article provide the body fluid type, clinical chemistry testing ordered, interference indices, and whether the indices exceeded the manufacturer's recommendations in the package insert for serum/blood specimens. The analyzed data are provided in the supplementary tables included in this article. The dataset reported is related to the research article entitled “Review of interference indices in body fluids specimens admitted for clinical chemistry analyses” [1].

**Table:** Specifications table

Subject	Medicine and Dentistry
Specific subject area	Pathology and Medical Technology
Type of data	Supplementary tables
How data were acquired	Body fluid and serum/plasma specimens analyzed by chemistry assays run on Roche Diagnostics cobas c502 and c702 clinical chemistry analyzers.
Data format	Raw and Analyzed
Parameters for data collection	Thirteen months of clinical chemistry analysis on body fluid specimens (February 1, 2017 to February 28, 2018) and one month of clinical chemistry analysis on serum/plasma specimens (February 1, 2018 to February 28, 2018), including interference indices, were extracted retrospectively from data in the electronic medical record.
Description of data collection	Data for a total of 2752 unique body fluid specimens and 25,507 unique serum/plasma specimens were obtained. This includes data for 12 different clinical chemistry assays (albumin, alkaline phosphatase, amylase, total bilirubin, blood urea nitrogen, total cholesterol, creatinine, glucose, lactate dehydrogenase, lipase, total protein, and triglyceride).
Data source location	Iowa City, Iowa, United States of America
Data accessibility	Raw data are available in this article as 2 Supplementary files.
Related research article	Author's name Renee L. Eigsti, Matthew D. Krasowski, Aditi Vidholia, Anna E. Merrill Title **Review of interference indices in body fluid specimens submitted for clinical chemistry analyses** Journal Practical Laboratory Medicine DOI https://doi.org/10.1016/j.plabm.2020.e00155

## Value of the Data

• The data provided are of value as there is currently only limited published data examining interference indices in body fluids submitted for clinical chemistry analyses.
• Other researchers or personnel in clinical laboratories might find these data useful as a reference for comparison.
• Our data set would serve as a starting point for researchers interested in future investigations examining the effects of hemolysis, icterus, and lipemia on body fluids analyzed by clinical chemistry assays marketed by vendors.
• The data are of value as previous studies have not examined a wide range of clinical chemistry assays with respect to body fluid analysis and interference indices.
• The data provide information for 2752 unique body fluid specimens and 25,507 serum/plasma specimens.

## 1 Data

We analyzed interference indices in body fluid and serum/plasma samples submitted for analysis at an academic medical center central clinical laboratory [1]. There is limited published data on interference indices in non-blood body fluids, and manufacturers of laboratory assays rarely provide data on the performance characteristics of body fluids for clinical chemistry assays [2], [3], [4], [5]. All data include patient age, legal gender in the electronic medical record, specific body fluid specimen, broader fluid category (abdominal fluid, cerebrospinal fluid, cyst contents, dialysate, drain fluids, gastric fluid, hepatic fluid, pancreatic fluid, pericardial fluid, pleural, respiratory secretions, stool, synovial fluid, urine, vaginal fluid, and wound exudate/fluid), quantitative hemolysis index, quantitative icteric index, quantitative lipemia index, clinical chemistry tests performed on the body fluid per clinician order and their quantitative analyte concentrations, and whether the interference indices exceed assay manufacturer thresholds in the assay package insert (which typically only reports interference limits for serum or plasma specimens). The raw data are included Supplementary file 1 (body fluids) and Supplementary file 2 (serum/plasma).

• Supplementary file 1: Data for 2752 unique body fluid specimens divided into categories**.** Specific data fields include: location/unit at time of testing (emergency department, inpatient, or outpatient), age in years, legal gender, exact body fluid type, category of body fluid, hemolysis index, icteric index, lipemia index, and quantitative analyte concentrations for 12 different clinical chemistry assays (albumin, alkaline phosphatase, amylase, total bilirubin, blood urea nitrogen, total cholesterol, creatinine, glucose, lactate dehydrogenase, lipase, total protein, and triglyceride). The data include the quantitative analyte concentration for the clinical chemistry test (or “Unable to report” if no analyte concentration could be obtained) and whether the interference index exceeded the parameter for that analyte for serum/plasma as reported in the manufacturer package insert.
• Supplementary file 2: Data for 25,507 unique serum/plasma specimens for 12 different clinical chemistry assays (albumin, alkaline phosphatase, amylase, total bilirubin, blood urea nitrogen, total cholesterol, creatinine, glucose, lactate dehydrogenase, lipase, total protein, and triglyceride). Specific data include hemolysis index, icteric index, lipemia index, and quantitative analyte concentrations for the different clinical chemistry assays. The data include the quantitative analyte concentration for the clinical chemistry test (or “Unable to report” if no analyte concentration could be obtained) and whether the interference index exceeded the parameter for that assay for serum/plasma as reported in the manufacturer package insert.

## 2 Experimental design, materials, and methods

All analyses were performed on Roche Diagnostics cobas 8000 analyzers (c502 and c702) as reported elsewhere [1,6]. The raw data consist of hemolysis index, icteric index, and lipemia index along with the specific clinical chemistry tests performed. Reporting Workbench functionality within the electronic medical record (Epic, Epic Inc., Madison, WI, USA) and Healthcare Enterprise Decision Intelligence (HEDI; an institutional data warehouse), along with data from middleware software (Instrument Manager version 8.14, Data Innovations, Burlington, VT, USA) were used to retrieve the data [7,8].

## Appendix Supplementary materials

Image, application 1
